# Coastal marshes provide valuable protection for coastal communities from storm-induced wave, flood, and structural loss in a changing climate

**DOI:** 10.1038/s41598-022-06850-z

**Published:** 2022-02-23

**Authors:** Y. Peter Sheng, Vladimir A. Paramygin, Adail A. Rivera-Nieves, Ruizhi Zou, Sarah Fernald, Timothy Hall, Klaus Jacob

**Affiliations:** 1 Engineering School of Sustainable Infrastructure and Environment, University of Florida, Gainesville, FL USA; 2 New York State Department of Environment Conservation and Hudson River National Estuarine Research Reserve, New York, NY USA; 3 The Climate Service, Durham, NC USA; 4 Lamont-Doherty Earth Observatory, Columbia University, Palisades, NY USA

**Keywords:** Climate sciences, Environmental sciences, Hydrology, Natural hazards, Mathematics and computing

## Abstract

Wetlands such as tidal marshes and mangroves are known to buffer coastal communities from wave, flood, and structural loss during storms. Coastal communities and resource managers seek to understand the ecosystem service value of coastal wetlands for reducing storm-induced flood loss in a changing climate. A recent modeling study found that a tall and dense *Phragmites*-dominated Piermont Marsh reduced the flood loss in the Village of Piermont, New York, U.S.A. during Superstorm Sandy and the 1% annual chance flood and wave event by 8% and 11%, respectively. Here we used the same modeling approach to examine the marsh’s buffering capacity in a changing climate (from 2020 to 2100), considering a potential marsh restoration plan (from 2020 to 2025) and potential marsh loss due to sea-level rise. Results showed that from 2020 to 2100, the 1% annual chance flood, wave, and structural loss would increase due to sea-level rise, storms, and marsh loss. However, the marsh will buffer ~ 11–12% of structural loss until 2050. Under the extreme SLR scenario of 2.89 m and a low accretion rate, Piermont Marsh is expected to lose its buffering capacity by 2080–2100 but will retain some buffering capacity with a high accretion rate of 10 mm/year and marsh growth. The marsh’s buffering capacity will remain during extra-tropical storms during winter and spring unless the wind has a significant northerly component. Lessons learned from this study can be used by coastal communities and marsh managers to develop coastal resiliency and marsh restoration plan.

## Introduction

Coastal wetlands such as tidal marshes and mangroves buffer coastal communities from wave, flood, and structural loss during tropical cyclones (TCs). As coastal communities strive to safeguard themselves from increasing storm-induced flood risks, they seek ways to maximize the protective powers of their natural features, such as coastal wetlands. However, the value of coastal wetlands for flood protection is often over-estimated^1,2^ or under-estimated^3^ due to flaws in the analysis. For example, Narayan et al.^1^, hereafter referred to as NAR17, over-estimated the value of wetland for flood protection in New Jersey (NJ) during Superstorm Sandy^4,5^, due to lack of model validation with the actual loss data, while Lathrop et al.^5,5^, herafter referred to as LAT19, under-estimated the wetland value due to the use of loss data and regression model only without a dynamic process-based flood model. Using historical data of combined wind and flood losses and regeression model only, Sun and Carlson^2^, herefafter referred to as SC20, grossly over-estimated the value of wetland for flood and wind protection. Over-estimation or under-estimation of the wetlands’ value for flood protection can greatly impact adaptation and resilience planning effort by coastal communities in the twenty-first century. Communities not only would like to know the value of coastal wetlands for flood protection in the current climate, they would also like to know the impact of climate change (e.g., sea level rise) as well as wetland management action on the value of coastal wetlands. Sheng et al.^6,7^, hereafter referredto as SHE21a and SHE21b, used extensive data and comprehensive dynamic and regression models to estimate the value of coastal wetlands for flood protection in NJ and New York (NY) under current climate condition. This paper extends the previous studies to quantify the value of coastal wetlands for flood protection in the twenty-first century (from 2020 to 2100) with various SLR scenarios and marsh management plans.

**Figure 1:**
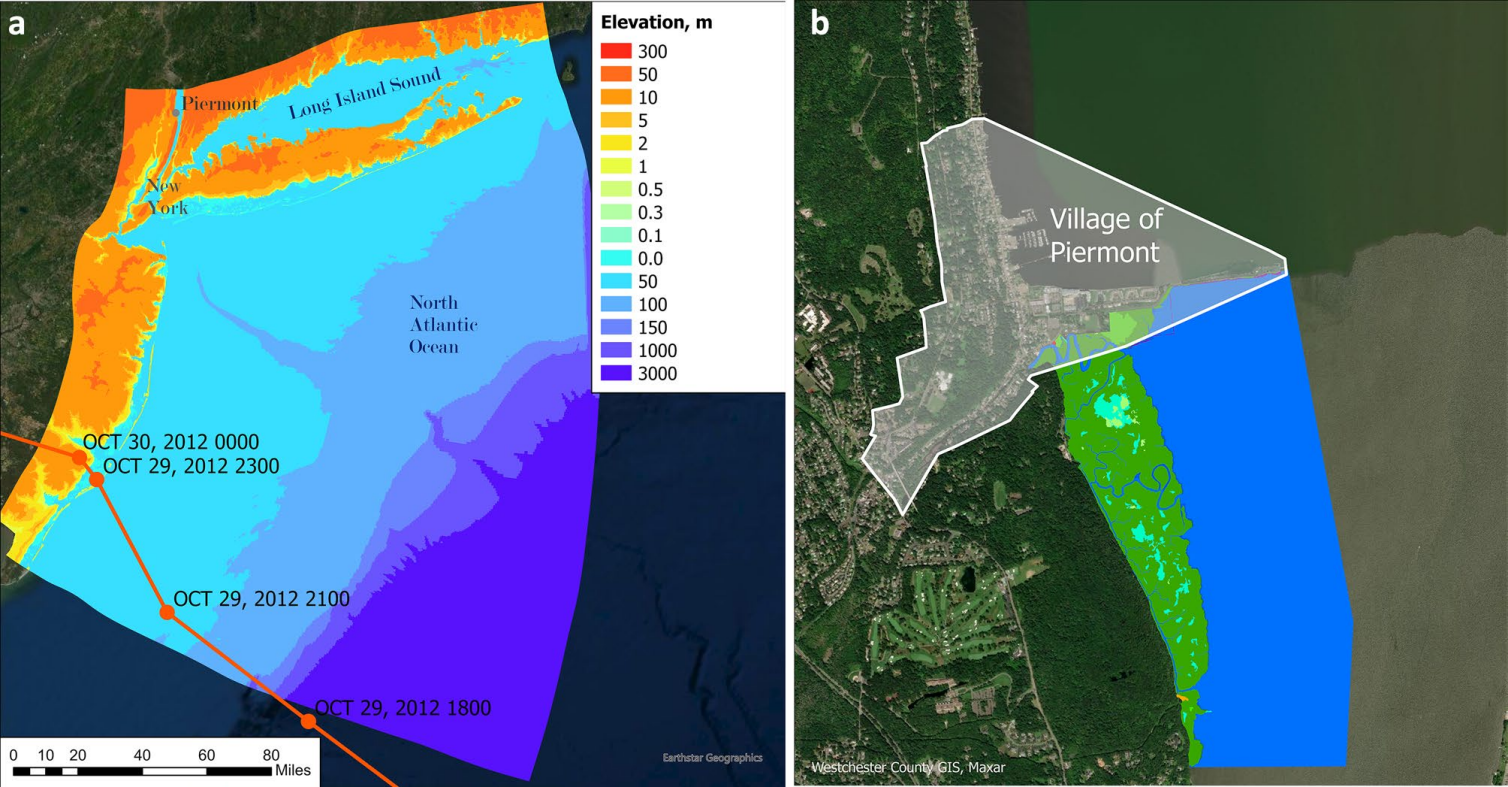
(**a**) NJ/NY/CT coastal domain and Hurricane Sandy (October 2012) track (left) (ArcGIS basemap credit: Earthstar Geographics). (**b**) Piermont Village and Marsh (right) (ArcGIS basemap credit: Westchester County GIS, Maxar).

During Superstorm Sandy, coastal wetlands along New Jersey (NJ), New York (NY), and Connecticut (CT) coasts (Fig. 1a) provided a modest reduction of structural loss in coastal communities^1,3,6^ due to the relatively sparse and low *Spartina* marsh and the high storm tide. On the other hand, Sheng et al.^7^ found that the tall and dense *Phragmites*-dominated Piermont Marsh buffered the Village of Piermont, located 40 km north (upstream) of New York City (NYC) on the Hudson River, from massive structural loss during Sandy. If the *Phragmites* were successfully replaced by the native *Typha* from the nearby Iona Marsh, the buffering capacity of Piermont Marsh was found to remain during Sandy unless it occurred in the winter or spring (an improbable occurrence for such high storm tides) when *Typha* is much lower and sparser.

The reduction of storm-induced flood damage in NYC by natural features varied geographically, and nature-based solutions to coastal flooding must be tailored to specific local conditions to be effective^8^. SHE21a^6^ and SHE21b^7^ found that the buffering capacity of coastal wetlands depends significantly on local wetland coverage and at-risk property value, as well as storm characteristics. Considering an ensemble of possible TCs^9^ for the NJ/NY/CT coasts, they found that the buffering capacity of coastal wetlands in NJ/NY/CT coasts^6^ and Piermont^7^ were able to provide higher buffering capacity (measured by percent reduction of potential structural loss) during the 1% flood and wave event, than that during Sandy.

With accelerating SLR and potentially more intense storms, the average wave crest (total flood elevation including storm surge and wave effect) is expected to increase between now and 2100, leading to increased structural loss unless the wetland is sustained or even increased, and at-risk properties are reduced. Previous studies, however, did not consider the effect of climate change and wetland restoration on the buffering capacity of coastal wetlands. While there is currently large uncertainty on projected TC activity at local spatial scales^10,11^, SLR is expected to accelerate^12–14^ and hence increase the flood damage to residential structures. It was predicted^15^ that Piermont Marsh would be inundated by SLR in 2080, assuming a low sediment accretion rate. Moreover, managers of the Piermont Marsh have proposed a potential phased restoration plan to replace 40 acres of *Phragmites* in the Piermont Marsh with *Typha* from the nearby Iona Marsh between 2020 and 2025. This study examines the effects of climate change, a potential marsh restoration plan, and potential marsh change on the ecosystem service value of Piermont Marsh for reducing storm-induced flood damage.

## Results

Maximum flood and significant wave height in Piermont during Sandy were simulated with and without the marsh^7^, SI Figs. 1, 2). While the two flood maps are comparable, the two wave maps are significantly different. Flood elevations simulated with and without the marsh are comparable, and flood elevation at the southern edge of the Village was about 99% of the flood elevation at the edge of the marsh in the Hudson River. On the other hand, with the marsh, wave height was 15-20 cm at the southern edge of the Village, compared to 60 cm at the marsh edge in the Hudson River due to rapid dissipation of the wave by the marsh within 150 m. Without the marsh, significant wave height at the southern Village would have been more than 50 cm, barely dissipated from the 60 cm at the marsh’s edge. Thus, while the maximum flood elevations during the 1% annual chance flood and wave are slightly higher than those during Sandy, the 1% wave heights are noticeably (~ 50%) higher than those during Sandy. See Supplemental Information (SI) for a detailed explanation. For a detailed explanation of the 1% annual chance flood and wave, see SI.

### Scenarios from 2020 to 2100 – considering TCs, SLR, and potential marsh restoration plan

**Figure 2:**
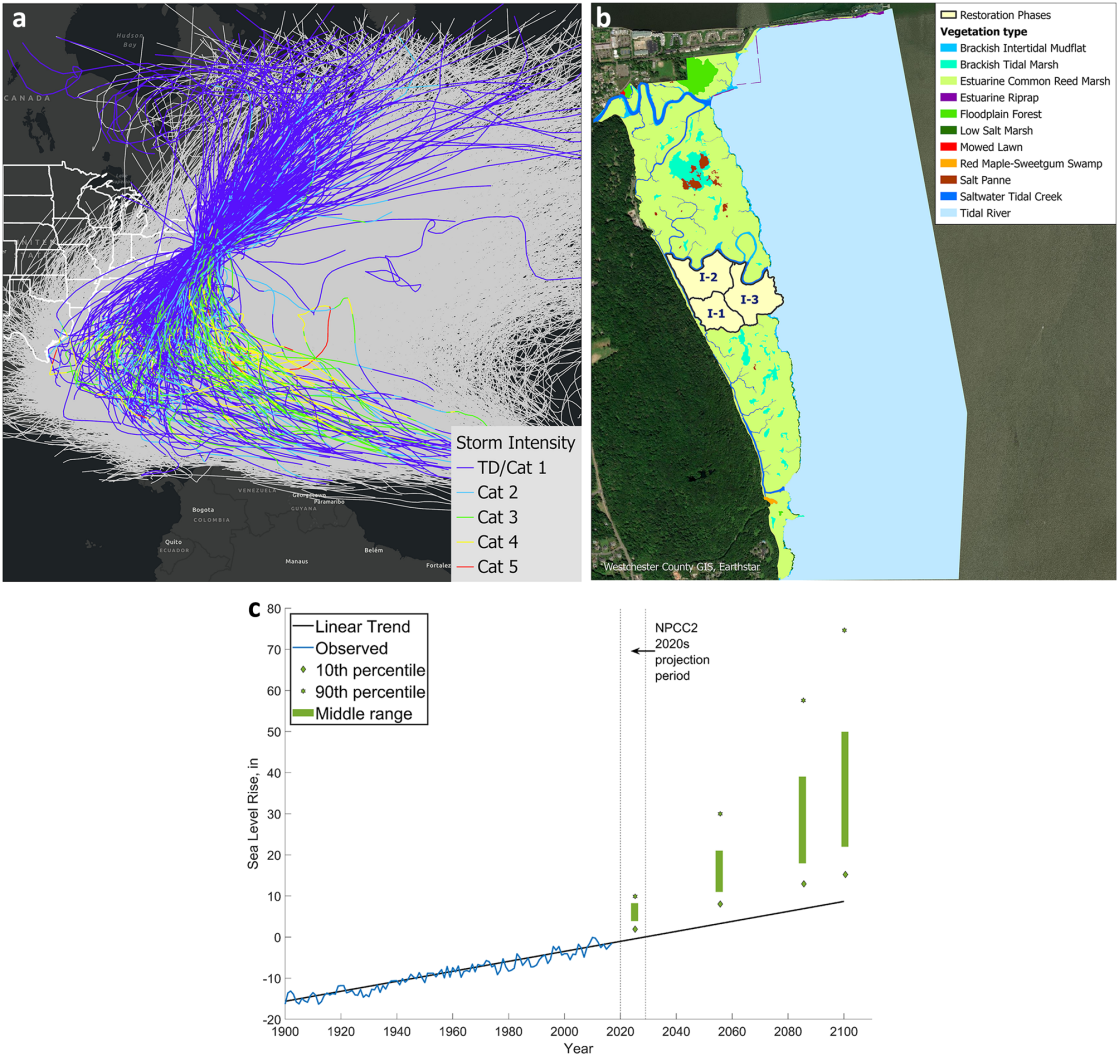
(**a**) Storm ensemble for NJ/NY/CT coasts predicted by the NASHM^11^ (upper left). (ArcGIS basemap credit: ESRI, HERE, Garmin, SafeGraph, FAO, METI/NASA, USPS, EPA, NPS). (**b**) Piermont Marsh with a 3-phase restoration plan to restore *Typha* in the 40-acre area (upper right). (ArcGIS basemap credit: Westchester County GIS, Maxar). (**c**) SLR at Battery^12^ (lower left)).

Table 1 lists six major scenarios considered for the Piermont area considering the effects of TCs, SLR, and marsh management plan. All scenarios used the TC ensemble (Fig. 2a) determined by the NASHM statistical hurricane model ^9,11,16^, which showed little projected change in TC activity on the US northeast through the 2030s. SLR values (Fig. 2c) are based on the New York Panel of Climate Change predictions^12^, Chapter 3, p.83, Table 3.2). The mean middle-range values of 6″ and 18″ are adopted for 2025 and 2050. For 2100 the extreme value of 114″, which corresponds to NPCC’s Arctic Rapid Ice Melt (ARIM) scenario^12^, is used. In addition, a potential 3-phase marsh restoration plan to restore three areas totaling 40 acres (Fig. 2b) from current condition (CC) to *Typha* between 2020 and 2025 was considered^17^. Details of the TC ensemble, SLR values, and the marsh restoration plan are described in the Methods section.

**Table 1:** Coastal flood scenarios from 2020 to 2100.

Scenario	Year	SLR (in)	SLR (cm)	Marsh restoration phase	Restoration area 1 (Fig. 3b)	Restoration area 2	Restoration area 3	Other marsh areas
1	2020	0	0	1	No Marsh	CC	CC	CC
2	2022	0	0	2	Low T	No Marsh	CC	CC
3	2025	6	15	3	High Typha	Low Typha	No Marsh	CC
4	2050	18	46	4	High Typha	High Typha	High Typha	CC
5	2050	18	46	–	CC	CC	CC	CC
6	2100	114	290	–	Marsh Lost	Marsh Lost	Marsh Lost	Marsh Lost

### 1% Annual chance flood and waves from 2020 to 2100

**Figure 3:**
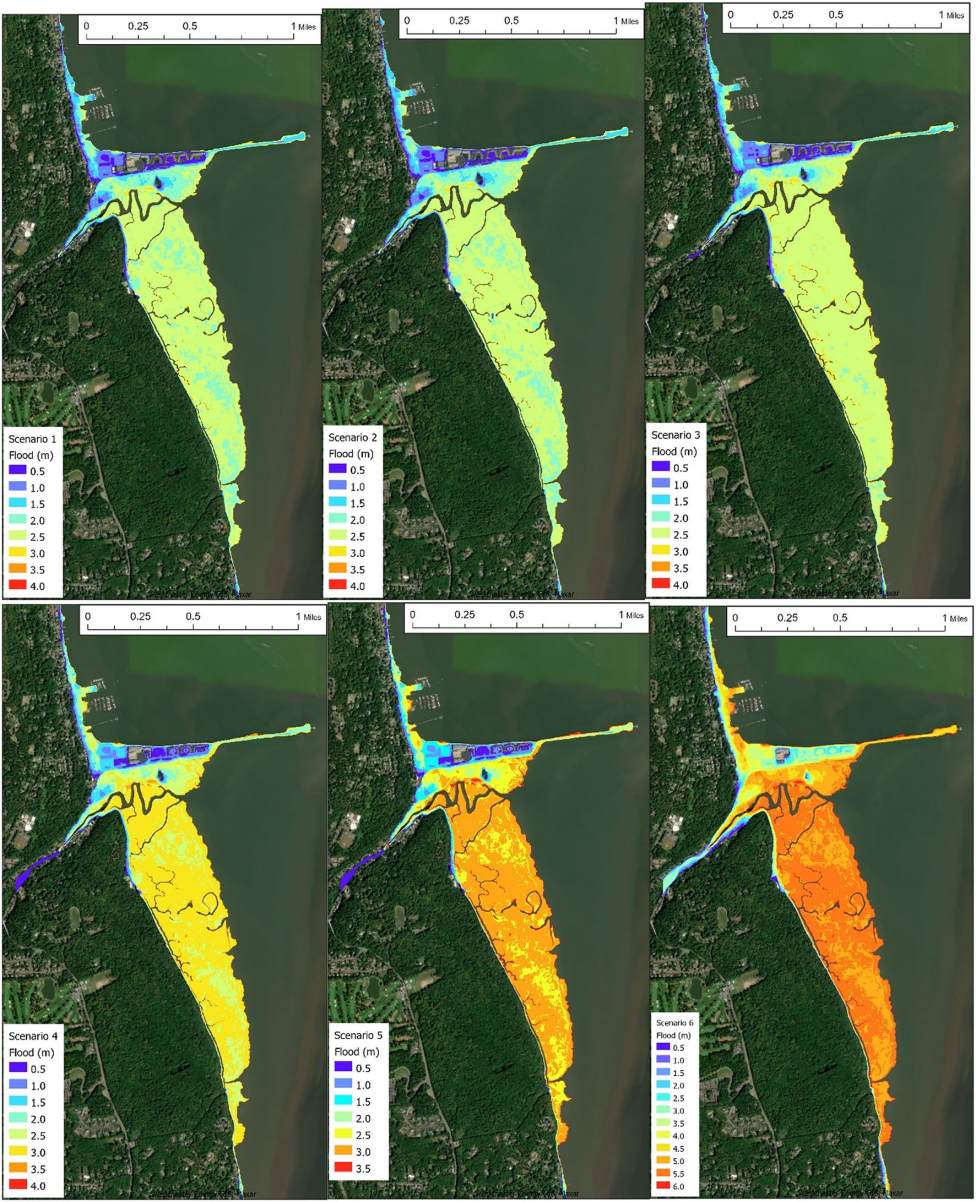
1% Annual chance flood maps of Piermont, NY for the six scenarios shown in Table 1. Top panel—Scenario 1,2,3 (left to right); lower panel—Scenario 4,5,6. (ArcGIS basemap credit: Westchester County GIS, Maxar).

As shown in Fig. 3, the 1% annual chance flood in Piermont shows that the flood at the marsh edge is comparable to the flood at the south Village, indicating the marsh had little effect in buffering flood. Flood elevation would change little from 2020 to 2025, suggesting that the proposed marsh restoration plan and the very mild SLR in 2025 would not impact the flood risk in the region. TCs remain the major cause of coastal flooding. Scenarios 4 and 5 show noticeably higher flood than the 2020–2025 period due to the significant (50th percentile) SLR value of 18″ (45 cm). More properties in the western Village would become inundated during a 1% event. Flood elevation for Scenario 4 (with fully restored *Typha* in the 40-acre area) and Scenario 5 (with current marsh condition) shows little difference, confirming that the modest marsh restoration plan would not change the Piermont Marsh buffering capacity. By 2100 (Scenario 6), however, due to the extremely high (ARIM) SLR value and the complete inundation of the marsh^15^, the marsh would lose its buffering capacity. Many properties in the western Village and on the pier would be under 6ft of floodwater.

**Figure 4:**
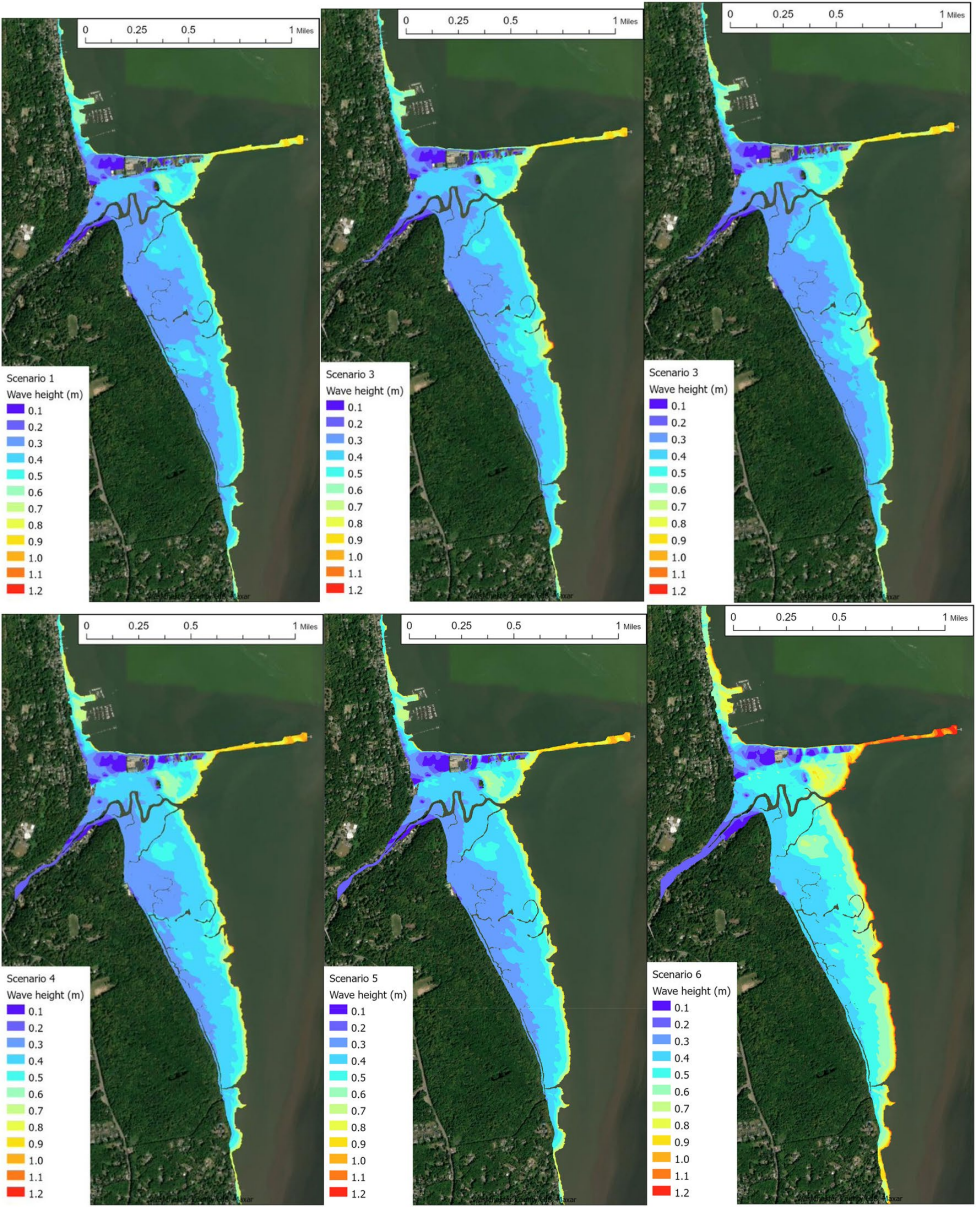
1% Annual chance significant wave height maps for Piermont, NY for the six scenarios shown in Table 1. Top panel—Scenario 1,2,3 (left to right); lower panel—Scenario 4,5,6. (ArcGIS basemap credit: Westchester County GIS, Maxar).

The 1% annual chance wave maps of Piermont (Fig. 4) indicate that the marsh would buffer the Village from wave damage by quickly dissipating the waves at the marsh edge with approximately 100 m. The wave maps exhibit the same temporal trend, with slight differences among Scenarios 1,2,3 but a significant increase for Scenarios 4 and 5 in 2050 and a dramatic increase for Scenario 6 in 2100. The increasing 1% flood elevation and wave height indicate that structural loss in Piermont Village will increase in the twenty-first century.

### Value of Piermont marsh for reducing flood-related structural damage in Piermont Village

#### Value of Piermont marsh during Sandy and 1% flood and wave

SHE21b^7^ simulated the maximum flood and wave in Piermont during Superstorm Sandy with and without the Piermont Marsh and found the marsh reduced the total structural loss by USD 0.9 M (7.6% of the estimated loss of USD 11.9 M with current marsh). For the 1% annual chance flood and wave event, the marsh reduced the total loss by USD 2.2 M (11.34% of the loss USD 18.8 M with marsh). These estimates were based on the 2018 property values of Piermont Village. Here we estimate the value of the Piermont marsh by dividing the avoided loss of USD 0.9 M and USD 2.2 M by the total flooded area (1.4 km^2^) of the marsh^18^, resulting in USD 0.64/m^2^ and 1.57/m^2^ for Sandy and the 1% event, respectively.

As shown by SHE21a^6^, the value of coastal wetlands for reducing flood- and wave-induced structural loss depends highly on the storm characteristics and local wetland and property conditions. Currently, there is no consensus on the best way to represent the value of coastal wetlands for flood protection. Here we present the scenario-dependent marsh value in terms of several metrics: total structural loss (TSL, in USD based on 2017 taxed property values), relative structural loss (RSL, in % of total property value), total avoided loss (TAL, as the difference between loss without marsh and loss with marsh), relative avoided loss (RAL, as TAL divided by the total property value), and unit marsh value (UMV, as TAL divided by the marsh area).

#### Value of Piermont marsh during Sandy and 1% flood and wave event from 2020 to 2100

**Figure 5:**
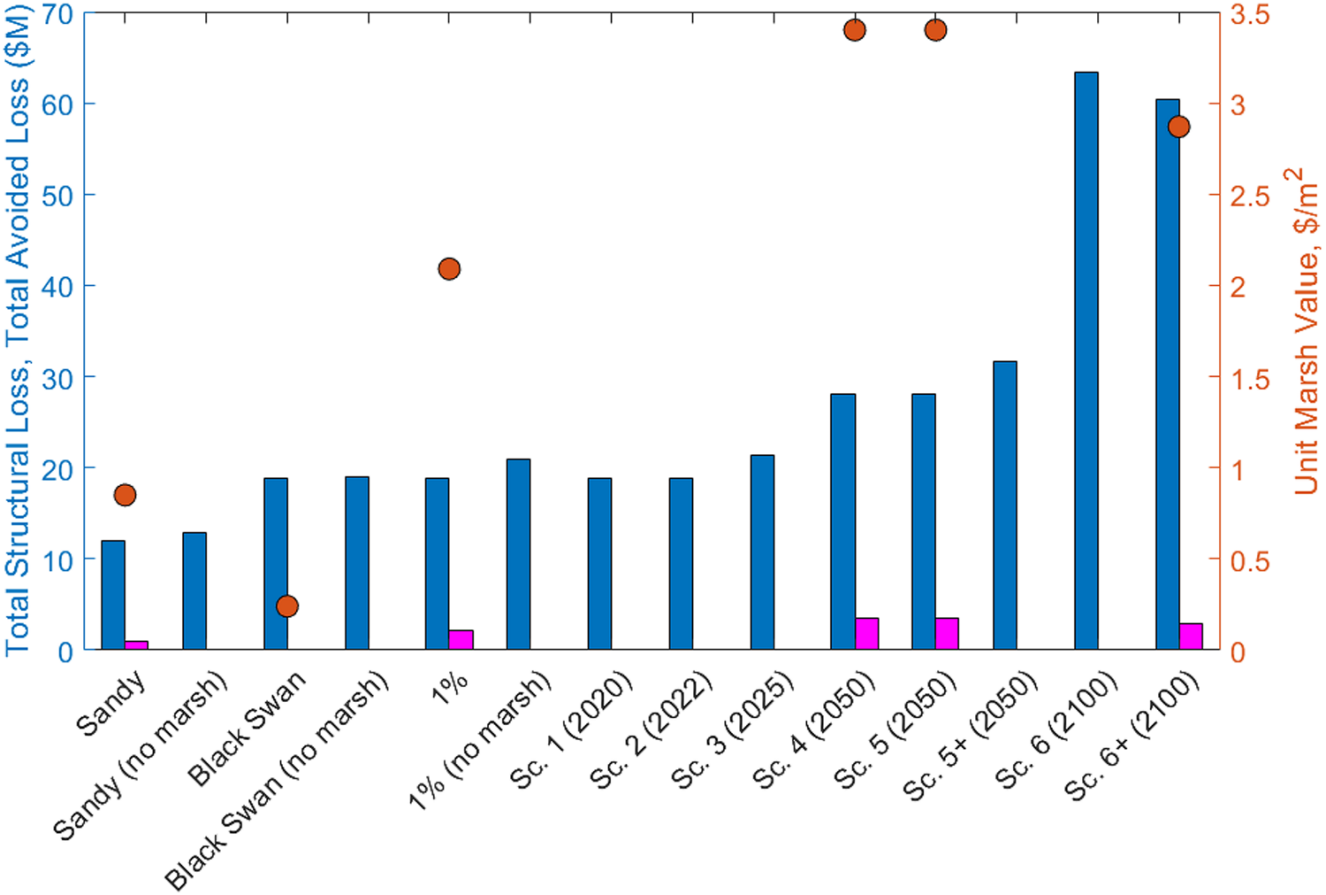
Total structural loss (blue bars), total avoided loss (magenta bars), and unit marsh value (orange circles) for scenarios are listed in Table 1.

The value of Piermont marsh for each scenario listed in SI Table 2, including Sandy, a rare “Black Swan” storm^6^, the 1% flooding, and the six scenarios are shown in Table 1 and plotted in Fig. 5. We also present the structural loss for several scenarios when the marsh is completely removed, including Sandy, Black Swan storm, and Scenario 5 in 2050. Scenario 6 represents the worst condition in that the marsh is entirely inundated by the marsh’s 2.89 m SLR and low accretion rate. Here we also include the results corresponding to a best-case Scenario 6, which assumes the high accretion rate of 12 mm/year and sufficient marsh growth to keep up with the extreme SLR.

#### Value of coastal wetlands in New Jersey

Aggregating the zip code scale results^6^ to county scale, the median value of coastal wetlands in all NJ counties is found to be 0.15, 5.91, 5.33, 4.82 USD/m^2^ for Sandy, Black Swan, 1% flooding in 2020, and 1% flooding in 2100. respectively. The corresponding values for Ocean County, NJ are 2.34, 2.55, 4.50, 2.05 USD/m^2^, respectively. These results show that the wetland value for flood protection depends on the TC, local property values, and wetland areas.

In comparison, the value of Piermont marsh is 0.85, 0.24, and 2.09 during Sandy, the Black Swan storm, and 1% flood and wave in 2020, respectively. The value of the Piermont marsh for the 1% flood and wave is 3.4 in 2050 and 2.79 in 2100.

**Figure 6:**
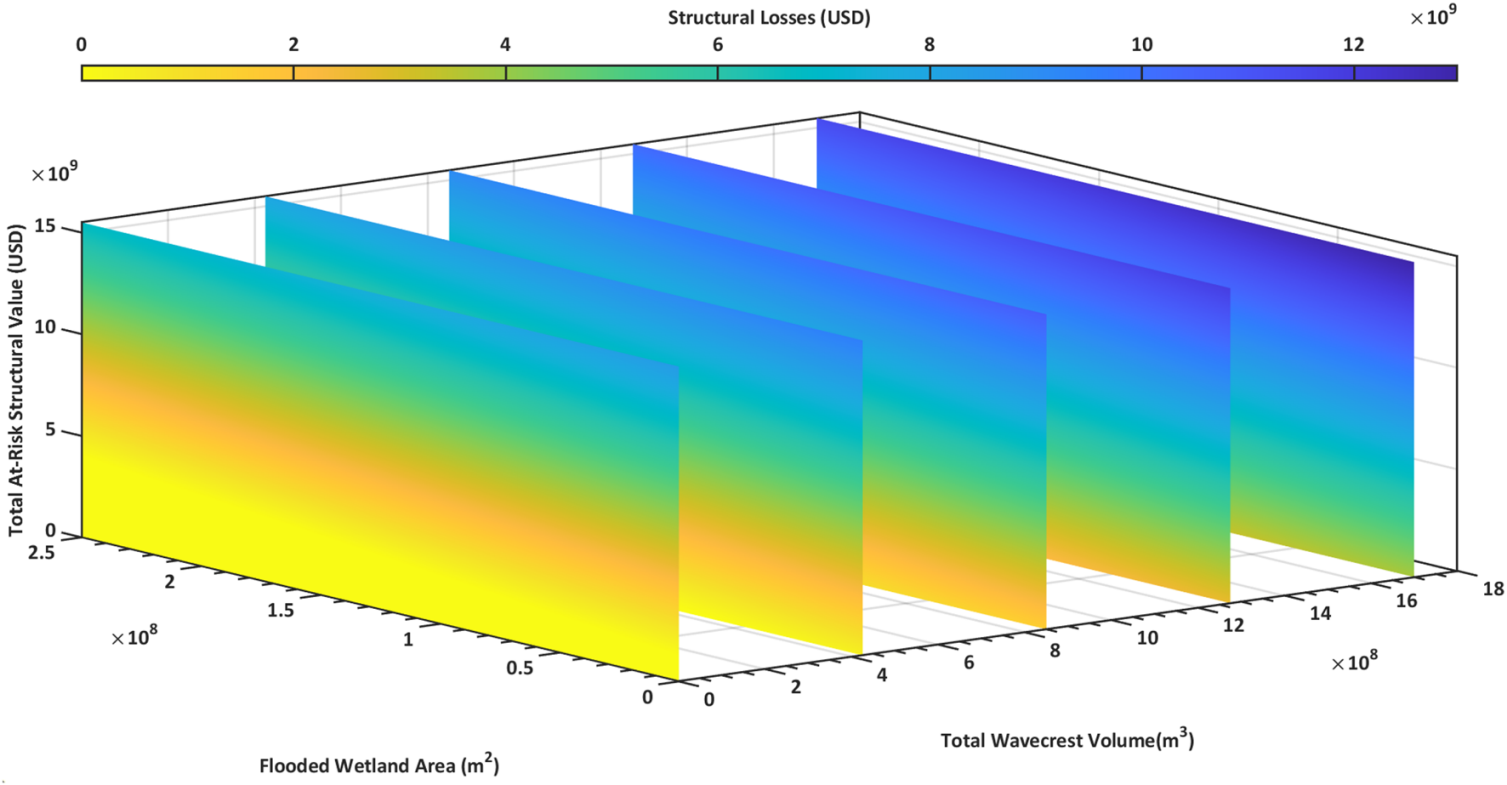
NJ county structural loss regression model. An ordinary least squared linear regression model. Constructed using the flooded wetland area, total at-risk structural value, and total wavecrest volume as predictors to estimate structural losses in zip-codes (R2 = 0.85, p = 2.78E-25).

To explain the significant spatial variation in structural loss reduction by wetlands, we developed an ordinary least square regression model fitted with data for all coastal counties during four flood events and with and without wetlands. The flooded wetland area, total at-risk structural value, and total wave crest volume in each county were used as predictors to estimate the structural losses in the county (SI Table 3). The regression model (Fig. 6) had a significant correlation with R^2^ = 0.85 and p = 2.78E-25. Losses in a county increase with the total at-risk structural value and the total wave crest volume but decreased with flooded wetland area. The absolute value of the t-statistic informs us that the most important feature is the total at-risk structural value followed by the total wavecrest volume, and lastly, the flooded wetland area.

## Discussion

Results show that Piermont marsh and coastal wetlands in NJ (on zip code scale) are highly dependent on the storm conditions (characterized by the total flood elevation or wave crest), local wetland condition, and at-risk property value. The wetland value for Sandy and the Black Swan storm differed significantly, suggesting strong dependence of wetland’s value on storm characteristics. Therefore, it is more appropriate to consider an ensemble of storms instead of a single design storm for meaningful valuation of coastal wetlands. The 1% flood elevation and wave height at any location represent the elevation exceeded by hundreds of TCs that have characteristics significantly different from Sandy or the Black Swan storm. During Sandy, due to the easterly peak wind, Piermont marsh could not dissipate surge but significantly dissipated the wave. However, during some storms in the ensemble, Piermont marsh could buffer the storm surge and the wave. Therefore, we use the 1% flood and wave as the benchmark condition for assessing the value of coastal wetlands in reducing flood- and wave-induced structural loss in a changing climate. The flood, wave, and structural losses associated with the 1% flood and wave increase over time. The 1% annual chance flood and wave event for 2020 may become a 10–20% annual chance event by 2100.

The study found that TCs will dominate the coastal flooding until 2050, when SLR takes over as the dominant driver. Thus, the marsh may be entirely inundated by the extreme SLR and lose its buffering capacity in 2100. However, recent findings of high accretion rate (> 10 mm/year) and continued growth associated with *Phragmites* marshes^19–21^ suggest that Piermont marsh could keep up with SLR and retain some buffering capacity. Therefore, the potential restoration plan to restore part of *Piermont* marsh with *Typha* during 2020–2025 would not affect the marsh’s buffering capacity.

The Unit Marsh Value (UMV) of Piermont marsh is estimated to increase from $2.09/m^2^ in 2020 to $3.4/m^2^ in 2050, due to a much more significant increase in TSL without the marsh than TSL with the marsh. For the best-case scenario in 2100, the Unit marsh value will remain at $2.79/m^2^. These values are lower than those for the coastal wetlands in New Jersey, estimated to be $5.33/m^2^ and $4.82/m^2^ for 2020 and 2100, respectively, due mainly to the higher property value in NJ. It should be noted that these estimated UMVs were based on the 2017 taxed property values. Should the property values change significantly in the future, the marsh value will also change accordingly. For example, if the property values were to triple by 2050, the UMV should also triple.

Due to lack of consensus on the valuation of coastal wetlands for reducing flood- and wave-induced residential structural loss, we present our economic analysis in terms of several metrics which measure the value of coastal wetlands. As shown in the following Table 1, Relative Avoided Loss (RAL) was used by NAR17^1^, LAT19^2^, and SHE21a^6^, and SHE21b^7^. Marginal wetland value, which appears to be comparable to the UMV (Unit Marsh Value) of this study, was used by SC20^2^. Notably, while NAR17^1^, SHE21a^6^ and SHE21b^7^ used a dynamic surge model and surge-wave model, respectively, to simulate the structural losses with and without wetlands, LAT19^3^ and SC20^2^ used linear regression analysis without any dynamic model results. The regression models of LAT19^3^ and SC20^2^ were constructed solely based on historical data but were not shown to have predictive ability. More explanations are given in the following paragraph

The linear regression models of LAT19^3^ and SC20^2^ did not take into consideration that total flood area, flooded wetland area, and total at risk property value have interactions. For example, LAT19^3^ suggested that the slope of the wetland width is the wetland value, while SC20^2^ developed a formula to calculate the wetland value. However, the assumptions made by them were too simplistic because they calculated the wetland value by replacing the flooded wetland areas with zero but used the same total at risk property value and total flood area corresponding to “with wetlands” results. Moreover, SC20^2^ performed log transformation on the predictors and the target of their regression in order to decrease the variability of the data and make the data to more closely resemble the normal distribution. However, the results of standard statistical tests achieved on log-transformed data are frequently not pertinent for the original, non-transformed data. As a result, these models fail to predict the correct total loss when replacing the total wetland area with zero.

We overcame the deficiencies of the regression models of LAT19^3^ and SC20^2^ by using validated dynamic model results to construct a regression model for prediction and inference purposes. Using the LAT19^3^ and SC20^2^ regression models, without the support of dynamic modeling results, will result in massive uncertainty in their predicted wetland values, e.g., the marginal values of wetland estimated by SC20^2^ for coastal counties in NJ and FL are 10–100 times of those predicted by our model. The more realistic values of coastal wetland for reducing flood- and wave-induced structural loss from our study can provide helpful information to coastal communities to develop coastal resiliency and wetland restoration plans.

## Methods

### A statistical tropical cyclone model: NASHM

The North Atlantic Stochastic Hurricane Model (NASHM), in combination with sea-surface temperature (SST) projections from climate models, was used to estimate regional changes in US TC activity into the 2030s^11^. NASHM is trained on historical variations in TC characteristics with two SST indices: 1, global-tropical mean SST and 2, the difference between tropical North-Atlantic SST and the rest of the global tropics, often referred to as “relative SST.” Testing confirms the model’s ability to reproduce historical US TC activity, as well as to make skillful predictions. When NASHM is driven by SST projections out to 2040, overall North Atlantic annual TC counts slightly increase, and the intensity distribution shifts to higher peak wind speeds. These increases are partially offset, however, by changing track patterns that reduce US east coast landfall probabilities per TC. As a result, projected changes in TC activity do not appear to significantly impact NY-region coastal flooding risk. Because of these projected modest changes in 2040 by NASHM, as well as the overall significant uncertainties in regional TC activity projections^10^, in this study, we assume the TC statistics remain unchanged between 2020 and 2100.

### A Coupled Hydrodynamic-Wave Model: CH3D-SSMS

CH3D-SSMS is an integrated storm surge modeling system^22–24^, incorporating a coastal surge model CH3D^25,26^ and a phase-averaged wave model SWAN^27^. CH3D-SSMS couples 2D/3D storm surge and wave models of coastal and basin-scale using a non-orthogonal curvilinear grid on the horizontal and a terrain-following sigma coordinate in the vertical for an accurate representation of estuaries and coastal waters. The coupled CH3D-SWAN coastal models receive open boundary conditions from the basin-scale surge model (ADCIRC^28^ or CH3D) and wave model (WWIII)^29^. The governing model equations and surge-wave coupling mechanisms of CH3D-SSMS are described elsewhere^22^ hence will not be repeated here.

CH3D uses the Reynolds-averaged Navier–Stokes (RANS) equations to compute water elevation and current velocities and represent the vegetation-induced drag forces to the mean flow and turbulence (Lewellen and Sheng 1980) by including extra profile drag $D_{P}$ and skin-friction drag $D_{S}$, which depend on the profile area ($A_{f}$) and wetted area ($A_{w}$) in the momentum Eqs.^7,23^. In the vegetation-resolving SWAN model^30^, vegetation is treated as cylinder units, and the plant-induced forces (drag and inertia forces) are modelled by accounting for irregular waves and depth-varying bottom, i.e., a vertical layer schematization for representing vegetation structure and calculating the dissipation term in the spectral action balance Eq.^31^. The drag coefficient and vertical profiles of $A_{f}$ and $A_{w}$ vary with the vegetation types (*Phragmites, Typha*, and mangrove)^7,24^. The robustness of the coupled CH3D-SWAN model for simulating the effects of vegetation on flow and wave has been verified with numerous laboratory and field experiments^6,7,24^. For simplicity and lack of detailed vegetation data in NJ, NY, and CT, SHE21a^6^ used the 2D vertically integrated version of CH3D.

### Regional scale and piermont scale model simulations

The study involves a regional NJ/NY/CT domain (Fig. 1) represented by a high-resolution curvilinear grid consisting of 1461 by 2182 cells with 40–50 m resolution in NYC and about 20 m in the low-lying land area, such as lower-Manhattan, to resolve the local complex geographic features. The grid domain covers the coasts of New Jersey, New York, and Connecticut. It also includes the Hudson River from the New York Harbor to the Federal Dam at Troy. It extends from the continental shelf to elevations that are higher than the possible extent of flooding by storm surge.

The two-dimensional version of CH3D-SWAN was run to simulate the surge and wave throughout the regional domain for each TC. In addition, the time series of water level along the boundary of the Piermont domain was used to drive the high resolution (4-5 m horizontal resolution with eight vertical layers) CH3D-SWAN model for the Piermont. Details of the regional scale study can be found in SHE21a^6^.

### The Joint Probability Method with Optimal Sampling (JPM-OS) statistical method

Given the storm ensemble generated by the NASHM model described in Sect. 3.1 and shown in Fig. 3a, we used JPM-OS^32,33^ to generate a set of optimal storms to represent all the possible storms described by the ensemble. We then simulated the 1% annual chance flood and significant wave height with and without the wetlands and produced the maps according to the following equations:

1 $$P\left[ {\eta_{max} > \eta } \right] = \lambda \smallint .{ }.{ }.\smallint f_{x} \left( x \right)P\left[ {\eta \left( x \right) > \eta } \right]dx$$

Here, $\lambda$ is the mean annual rate for all storms on the site, $f_{x} \left( x \right)$ is the joint probability density function of the storm characteristics, and *P* is the conditional probability that a storm with specific characteristics $x$ will cause a water level height or significant wave height above $\eta$. This integral is evaluated for every possible combination of storm characteristics. In practice, since the integral in Eq. (1) is not easily determined, it is usually estimated as a weighted sum of discrete storm parameters value.

2 $$P\left[ {\eta_{max} > \eta } \right] = \mathop \sum \limits_{i = 1}^{n} \lambda_{i} P[\eta \left( {x_{i} } \right) > \eta ]$$

Using the simulated coastal flood elevations for all the optimal storms, the probabilistic flood elevation and wave height at every grid cell of any desired return period, e.g., 100 years (1%) or 500 years (0.2%), can be calculated by the JPM-OS.

### Economic analysis

As a first step to assess the ecosystem service value of the Piermont Marsh in reducing property damage due to flood and waves during storms, we conducted a parcel-based economic analysis using the simulated inundation and waves along with the best available building footprint data^34^ and damage functions^35^. The height of the wave crest curve was used in regions where the depth limited controlling wave height $H_{C}$ was greater than 1.5 ft, while flood depth curve was used elsewhere. Here, wave crest (*WC*) is defined as:

3 $$WC = 0.7*H_{c} + d$$

Depending on the location of a property, different formulas with damage functions determined in the USACE study can be used to calculate the structural loss (SL). The total structural loss (TSL) is the sum of SL for all residential structures. The Relative Structural Loss (RSL) is the TAL as a percent of the total property value, which, in this study, is based on the 2017 taxed value of the properties.

To assess the value of coastal wetlands for reducing flood- and wave-induced structural loss, we introduce the following metrics: Total Avoided Loss (TAL), Relative Avoided Loss (RAL), and Unit Marsh Value (UML) for the Piermont Marsh for all scenarios. TAL is the difference between the TSL without marsh subtracted by the TSL with the marsh. RAL is the ratio between TAL and TSL with the marsh. Finally, unit Marsh Value is defined as the TAL divided by the total marsh area. Importantly, this study used the simulation results of dynamic models as input for the economic analysis, verified by comparison with the FEMA NFIP payout data in New Jersey^6^ and Piermont Village^7^.

### Schematics of the study

**Figure 7:**
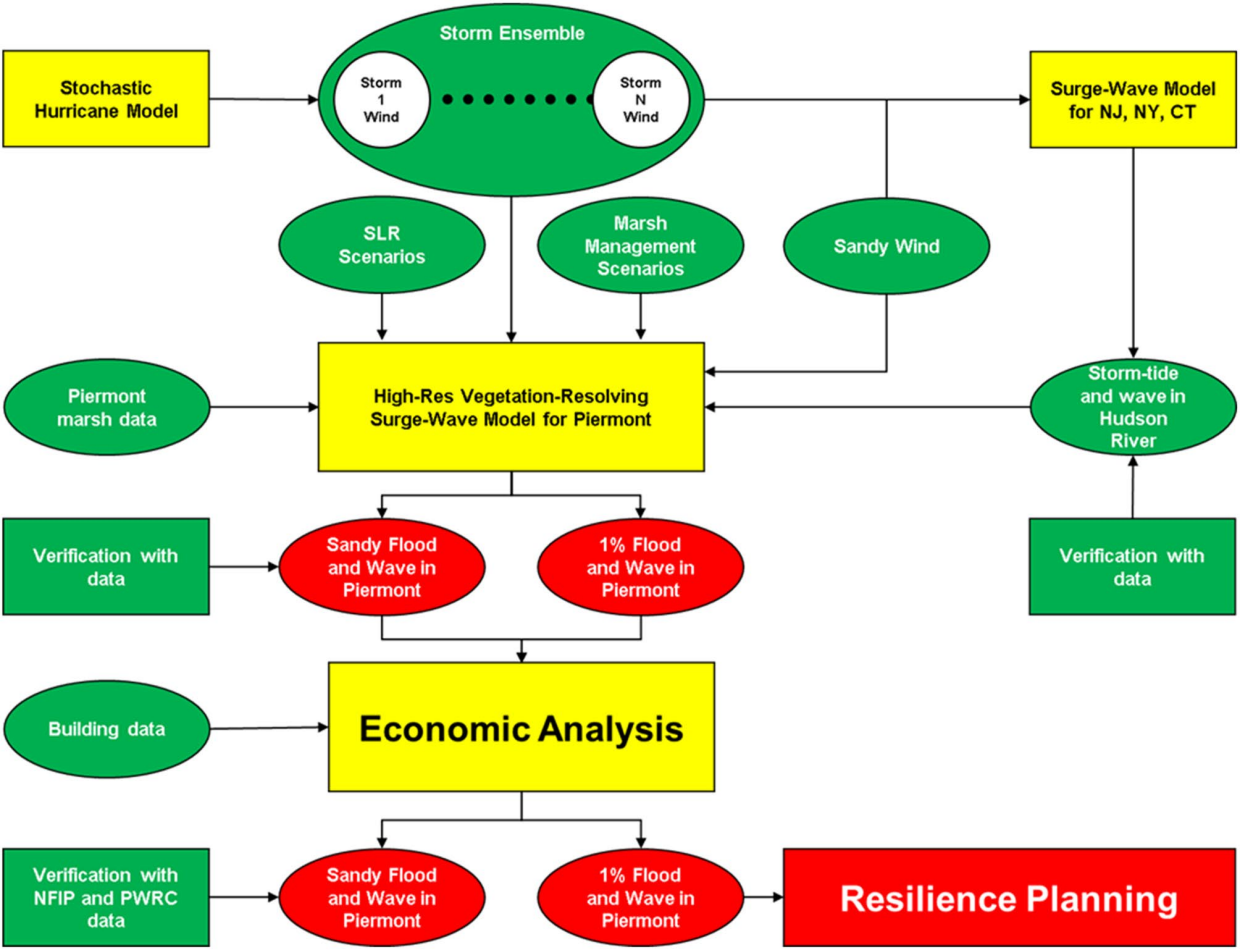
Schematics of the overall study.

This study focuses on the simulation of surge and wave in the Piermont Marsh and Village during 1% flood and wave events from 2020 to 2100. The overall study design is shown in Fig. 7. The three-dimensional vegetation-resolving CH3D-SWAN was used to simulate the surge and wave during Sandy and an ensemble of storms (predicted by the NASHM model) in a large regional NJ/NY/CT domain which includes the Piermont Marsh and Village. Model simulated water level along the boundary of the high-resolution Piermont domain was used to force the Piermont model. The simulated flood and wave in the Piermont domain were then used to conduct an economic analysis to estimate the structural loss of the residential properties. Simulations were conducted with the same storm ensemble but different SLR values involving different configurations of the Piermont marsh: 1) existing Phragmites-dominant Piermont Marsh, 2) Piermont Marsh removed, and 3) *Phragmites* replaced by *Typha* during three phases of the potential marsh restoration plan. These simulation results were used for the economic analysis described in the main body of the paper. Details of the regional-scale simulation and model results can be found in SHE21a^6^.

## Supplementary Information

Supplementary Information.

## References

[1] Narayan S, Beck MW, Wilson P, Thomas CJ, Guerrero A, Shepard CC, Reguero BG, Franco G, Ingram JC, Trespalacios D (2017). The value of Coastal Wetlands for flood damage reduction in the Northeastern USA. npj Sci. Rep..

[2] Sun F, Carson RT (2020). Coastal wetlands reduce property damage during tropical cyclones. Proc. Natl. Acad. Sci..

[3] Lathrop, R. G., Irving, W., Seneca, J. J., Trimble, J. & Sacatelli, R. M. The limited role salt marshes may have in buffering extreme storm surge events: Case study on the New Jersey shore, *Ocean Coast. Manag.* **178** (2019).

[4] Blake, E. S., Kimberlain, T. B., Berg, R. J., Cangialosi, J. P. & Beven, II J. L. Tropical Cyclone Report Hurricane Sandy (AL182012) 22–29 October 2012, National Oceanic and Atmospheric Administration – National Hurricane Center (2013).

[5] Federal Emergency Management Agency (FEMA). Hurricane Sandy in New Jersey and New York: Building performance observations, recommendations, and technical guidance. *Mitig. Assess. Team Rep.* **223**, (2013).

[6] Sheng YP, Rivera-Nieves A, Zou R, Paramygin V (2021). Role of wetlands in reducing structural loss is highly dependent on characteristics of storms and local wetland and structure conditions. Npj Sci. Rep..

[7] Sheng YP, Rivera-Nieves A, Zou R, Paramygin V, Angelini C, Sharp S (2021). Invasive Phragmites provides superior wave and surge damage protection relative to native plants during storms. Environ. Res. Lett..

[8] Wong SM, Gurian PL, Daley J, Bostrom H, Matsil M, Montalto FA (2020). A preliminary assessment of coastal GI’s role during Hurricane Sandy: A case study of three communities. Urban Water Journal.

[9] Hall T, Yonekura E (2013). North American tropical cyclone landfall and SST: A statistical model study. J. Clim..

[10] Knutson T (2020). Tropical cyclones and climate change assessment, Part II: Projected response to anthropogenic warming. Bull. Amer. Meteor. Soc..

[11] Hall TM, Kossin JP, Thompson T, McMahon J (2021). US tropical cyclone activity in the 2030s based on projected changes in tropical sea-surface temperature. J. Climate.

[12] NPCC 2019, New York City Panel on Climate Change 2019 Report, Chapter 3: Sea Level Rise. Ann. N.Y. Acad. Sci. ISSN 0077–8923. https://nyaspubs.onlinelibrary.wiley.com/doi/epdf/, 10.1111/nyas.14006 (last accessed, 6/21/2021). 10.1111/nyas.14006 30875120

[13] Kopp RE, DeConto RM, Bader DA, Hay CC, Horton RM, Kulp S, Oppenheimer M, Pollard D, Strauss BH (2017). Evolving understanding of antarctic ice-sheet physics and ambiguity in probabilistic sea-level projections. Earth’s Future.

[14] Sweet, W.V., Kopp, R.E., Weaver, C.P., Obeysekera, J., Horton, R.M., Thieler, E.R., & Zervas, C. *Global and Regional Sea Level Rise Scenarios for the United States*. *NOAA Technical Report NOS CO-OPS 083. NOAA/NOS Center for Operational Oceanographic Products and Services *(2017).

[15] Tabak NM, Laba M, Spector S (2016). Simulating the effects of sea level rise on the resilience and migration of Tidal Wetlands along the Hudson River. PLoS ONE.

[16] Hall TM, Jewson S (2007). Statistical modelling of North Atlantic tropical cyclone tracks Tellus. Ser. A Dyn. Meteorol. Oceanogr..

[17] New York State Department of Environmental Conservation (NYSDEC) (2019). Hudson River National Estuarine Research Reserve Management Plan.

[18] Yozo, D. J. *et al.* Ecological Profile of the Hudson River Estuarine Research Reserve. *NYSDEC Contract Report C004646*. 165 pp (2005).

[19] Rooth JE, Stevenson JC (2000). Sediment deposition patterns in Phragmites australis communities: Implications for coastal areas threatened by rising sea-level. Wetl. Ecol. Manag..

[20] New Jersey Department of Environmental Protection Science Advisory Board (NJDEP-SAB). *The Status and Future of Tidal Marshes in New Jersey Faced with Sea Level Rise*. 68 pp (2020).

[21] Weis JS, Watson EB, Ravit B, Harman C, Yepsen M (2021). The status and future of tidal marshes in New Jersey faced with sea level rise. Anthropocene Coasts.

[22] Sheng YP, Alymov V, Paramygin VA (2010). Simulation of storm surge, wave, currents, and inundation in the outer banks and Chesapeake bay during Hurricane Isabel in 2003: The importance of waves. J. Geophys. Res. Ocean..

[23] Sheng YP, Lapetina A, Ma G (2012). The reduction of storm surge by vegetation canopies: Three-dimensional simulations Geophys. Res. Lett..

[24] Sheng YP, Zou R (2017). Assessing the role of mangrove forest in reducing coastal inundation during major hurricanes. Hydrobiologia.

[25] Sheng Y.P. A Three-dimensional mathematical model of coastal, estuarine and lake currents using boundary-fitted grid (1986)

[26] Sheng Y. P. Evolution of a Three-dimensional curvilinear-grid hydrodynamic model for estuaries, lakes and coastal waters: CH3D Estuar. *Coast. Model.* 40–49 (1989)

[27] Booij N, Ris RC, Holthuijsen LH (1999). A third-generation wave model for coastal regions:1. Model description and validation. J. Geophys. Res. Ocean..

[28] Luettich, R. A., Westerink, J. J. & Scheffner, N. W. ADCIRC: An Advanced Three-Dimensional Circulation Model for Shelves Coasts and Estuaries, Report 1: Theory and Methodology of ADCIRC-2DDI and ADCIRC-3DL, Dredging Research Program Technical Report DRP-92–6. Dredging Research Program Technical Report DRP-92–6, U.S. Army Engineers Waterways Experiment Station, Vicksburg, MS (1992).

[29] Tolman, H. L. *et al.* User manual and system documentation of WAVEWATCH III (R) version 5.16. National Oceanic and Atmospheric Administration, National Center for Environmental Prediction. 310 pp (2016).

[30] Suzuki T, Zijlema M, Burger B, Meijer MC, Narayan S (2012). Wave dissipation by vegetation with layer schematization in SWAN. Coast Eng..

[31] Mendez FJ, Losada IJ (2004). An empirical model to estimate the propagation of random breaking and nonbreaking waves over vegetation fields. Coast Eng..

[32] Condon AJ, Sheng YP (2012). Optimal storm generation for evaluation of the storm surge inundation threat. Ocean Eng..

[33] Yang K, Paramygin VA, Sheng YP (2019). An objective and efficient method for estimating probabilistic coastal inundation hazards. Nat. Hazards.

[34] NYS GIS Clearinghouse - NYS GIS Program Office New York State Tax Parcels Online: http://gis.ny.gov/gisdata/inventories/details.cfm?DSID=1300

[35] U.S. Army Corps of Engineers (USACE) 2015 Physical Depth Damage Function Summary Report, North Atlantic Comprehensive Coastal Study: Resilient Adaptation to Increasing Risk Online: http://www.nad.usace.army.mil/Portals/40/docs/NACCS/10A_PhysicalDepthDmgFxSummary_26Jan2015.pdf

[36] FEMA Offical Flood Map, https://msc.fema.gov/portal/home (accessed January 23, 2021).

[37] Nadalaballo NC, Melby JA, Gonzalez VM (2015). Statistical analysis of historical extreme water levels for the U.S. North Atlantic Coast using Monte Carlo life-cycle simulation. J Coast Res.

[38] Village of Piermont (2014). Resilience Roadmap: Planning for Piermont’S Future.

