# Supplementary material for: Coastal marshes provide valuable protection for coastal communities from storm-induced wave, flood, and structural loss in a changing climate
Source: Sci Rep. 2022 Feb 23;12:3051. doi: 10.1038/s41598-022-06850-z (PMC8866407; doi:10.1038/s41598-022-06850-z)
Supplement: Supplementary file 1 — Supplementary Information. [file 41598_2022_6850_MOESM1_ESM.docx]

Supplemental Information

Maximum flood and wave during Superstorm Sandy

The maximum flood map and wave map during Superstorm Sandy are shown in the left panel of Figures 1 and 2, respectively. The maximum flood map and wave map during Sandy with the marsh removed are shown in the middle panel of Figures 1 and 2, respectively. While the two flood maps are comparable, the two wave maps are significantly different. Flood elevations obtained with and without the marsh are comparable, and flood elevation at the southern edge of the Village was about 99% of the flood elevation at the edge of the marsh in the Hudson River. On the other hand, with the marsh, wave height was 15-20cm at the southern edge of the Village, compared to 60cm at the marsh edge in the Hudson River due to rapid dissipation of the wave by the marsh. Details of the mechanism and simulation of wave dissipation by the marsh can be found in SHE21b^7^. Without the marsh, significant wave height at the southern Village would have been more than 50 cm, barely dissipated from the wave height at the edge of the marsh. Within about 150 m, the wave height was dissipated by more than 50%, consistent with the dissipation length shown in Equation (5) of SHE21b^7^.

Maximum flood and wave during the 1% annual chance event

Maximum flood and wave maps during a 1% annual chance event are shown in the right panel of Figures 1 and 2. While the maximum flood elevations during the 1% annual chance event are slightly higher than those during Sandy, the maximum wave heights during the 1% event are noticeably (~50%) higher than those during Sandy. This can be understood as explained below. The maximum flood and wave during Sandy were generated by the specific wind conditions during Sandy, which included strong southeasterly wind during the peak of Sandy. However, the 1% maximum flood and wave were generated by an ensemble of (a few hundred) generally less intense but more frequent TCs compared to Sandy. These different TCs are associated with winds of different speeds and directions. These winds with different directions do not result in significantly higher flood elevations because winds from certain directions cannot generate enough flood due to the relatively short fetches. However, winds from different directions can generate high wave elevations even with relatively short fetches.

Determination of the 1% annual chance floor and wave and comparison with others

1) For any given location, the simulated flood elevations from various storms, each with a distinct storm rate, are ranked from the highest to the lowest.

2) Starting from the top-ranked storm which yields the highest flood elevation, corresponding storms rates are added in descending order of the flood elevation until the sum of storm rates reaches a desired percent, e.g., 1%. The flood elevation corresponding to the last storm in the group is denoted the 1% annual chance flood elevation.

As an example, at the Battery of NYC, a total of 258 storms were found to be in the group of storms which yielded a 1% flood elevation of 12.9 ft. The storm rate in the group of storms ranged from 0.00000451 to 0.00012911, with an average storm rate of 0.000038759 (which is basically 1% or 0.01 divided by 258). These numbers can also be largely affected by the size of the storm ensemble and are not universal.

As shown in Table 1 below, the 1% flood are in reasonable agreement with those from the official FEMA flood map^37^ (<https://msc.fema.gov/portal/home>, acccessed January 23, 2021), our values are generally slightly lower than those predicted by FEMA, but are generally within 1.5 ft (with FEMA BFE values being integers). There are outliers but often it’s due to FEMA maps using artificially produced integer contour lines (often two lines next to each other can be different by 2 ft or more). On the other hand, 1% flood elevations determined by Nadal-Caraballo et al. (2015)^38^ of U.S. Army Corps of Engineers (USACE) are generally 25-50% lower than the values determined by this study and FEMA due most likely to their inclusion of many extra-tropical storms which generate much lower storm surge.

| Location | USACE | UF | FEMA |
| --- | --- | --- | --- |
| New London, CT | 7.6 | 10.6 | 11 |
| Montauk, NY | 6.5 | 11.1 | 12 |
| Kings Point, NY | 12.6 | 15.6 | 17 |
| The Battery, NY | 8.0 | 12.9 | 14 |
| Sandy Hook, NJ | 8.8 | 12.8 | n/a |
| Atlantic City, NJ | 6.8 | 13.1 | 14 |

Table 1. 1% flood elevation (ft) at selected locations obtained by UF, USACE, and FEMA.

Value of Marsh for reducing structural loss due to flood & wave during Sandy

As shown in SHE21b^7^, flood and wave during Sandy resulted in $11.9M structural loss in the Village, which compared well with the $20M (which includes additional loss associated with commercial properties and marinas, etc.) estimated by the Village of Piermont (2014)^39^. 70% of the loss was due to flood while 30% was due to wave. When Piermont marsh was removed, the model estimated structural loss increased to $12.8M. The marsh reduced the total structural loss by $902K (7.6% of the total actual loss, including 0.03% of flood loss and 26.2% of wave loss).

Value of marsh for reducing structural loss due to flood & wave during 1% annual chance event

SHE21b^7^ showed flood and wave during 1% event resulted in $18.8M structural loss in the Village, consisting of $11.1M flood-related loss and $7.69M wave-related loss. When Piermont marsh was removed, the model estimated structural loss increased to $21.0M ($12.3M flood loss and $8.62M wave loss). However, the marsh reduced the total structural loss by $2.13M (11.34% of the total loss with marsh, including 10.8% of flood loss and 12.1% of wave loss). Thus, the relative value of the marsh for reducing flood- and wave-induced structural loss is higher during the 1% flood and wave event than Sandy.


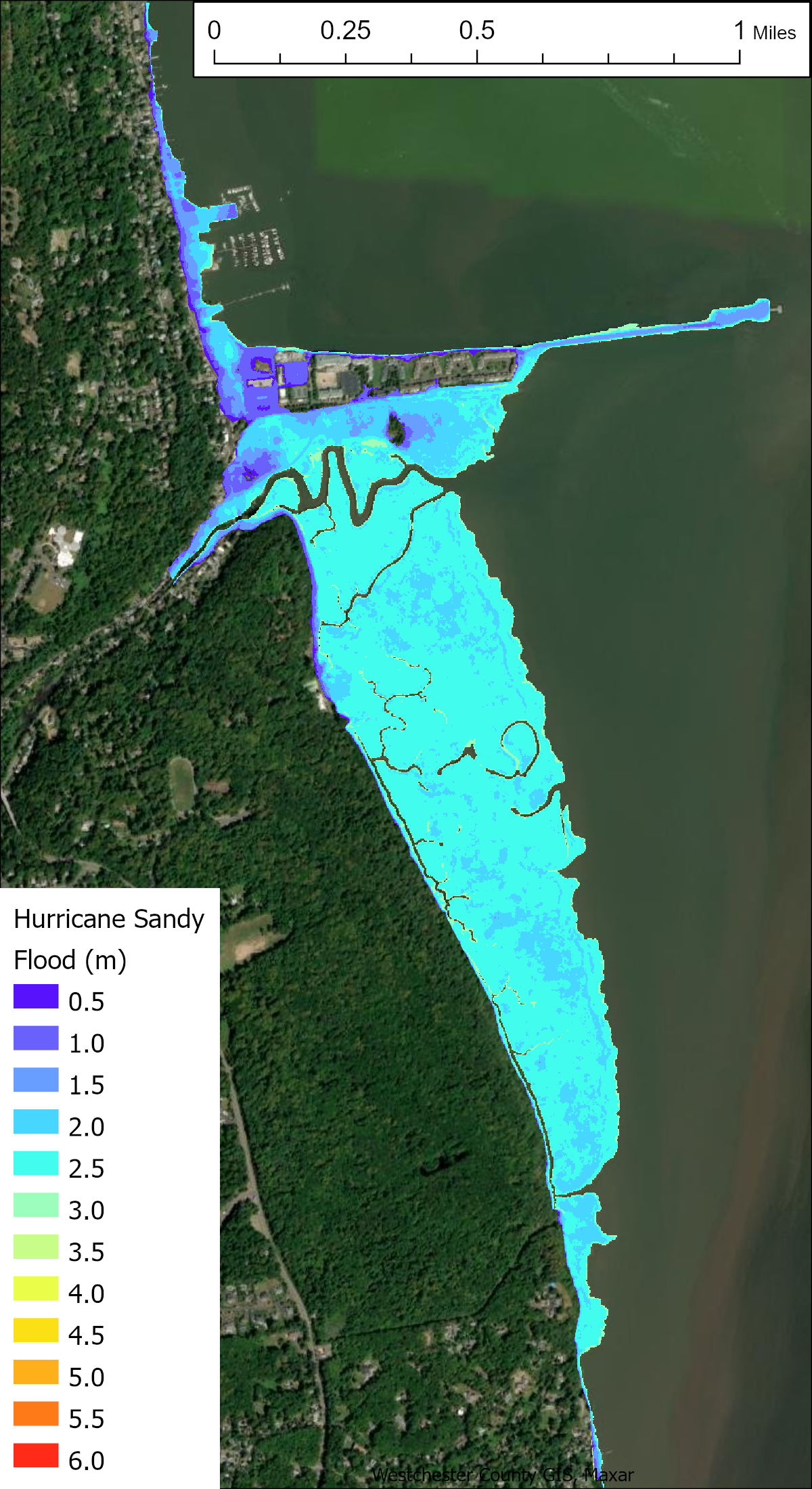

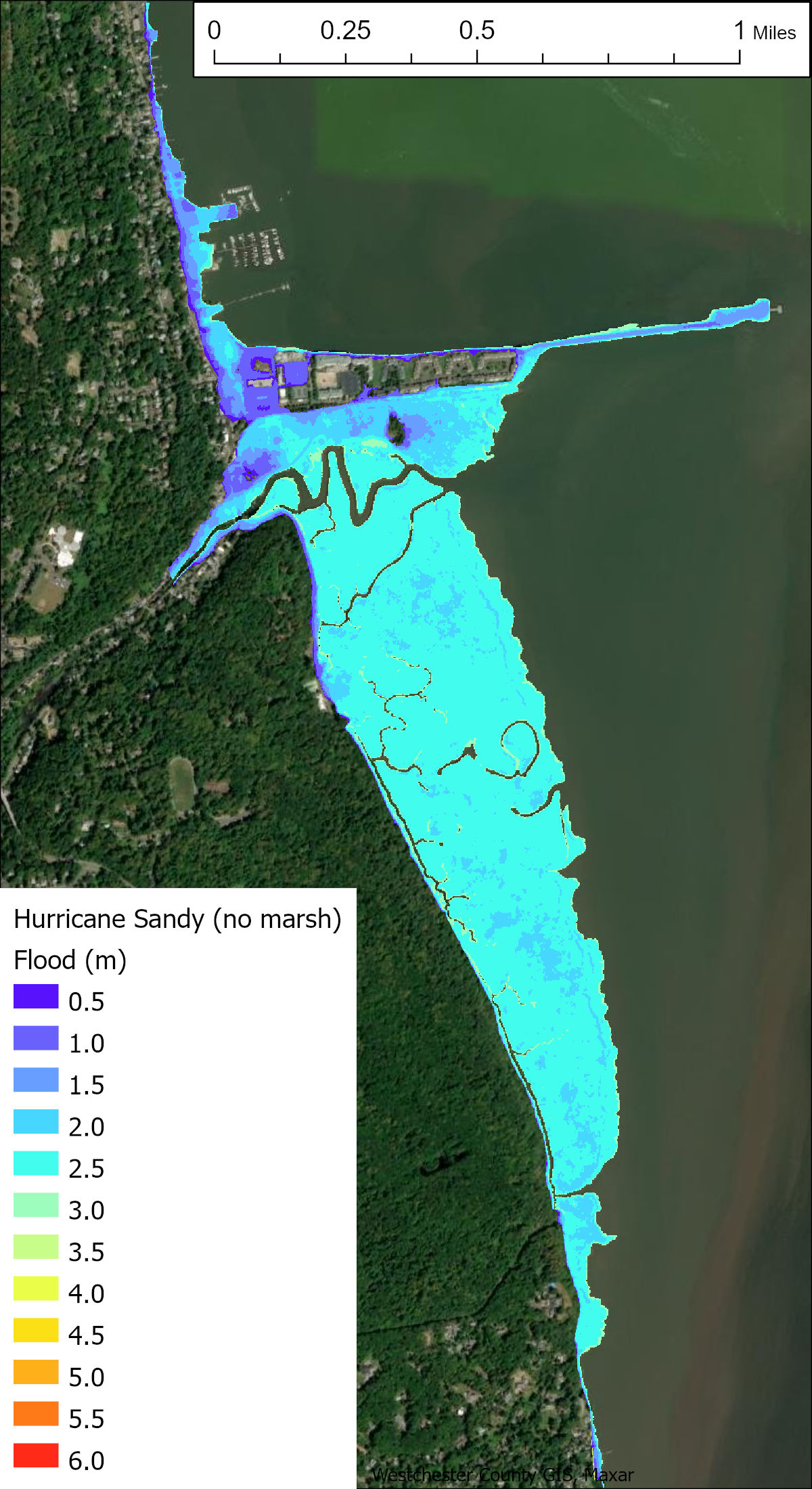

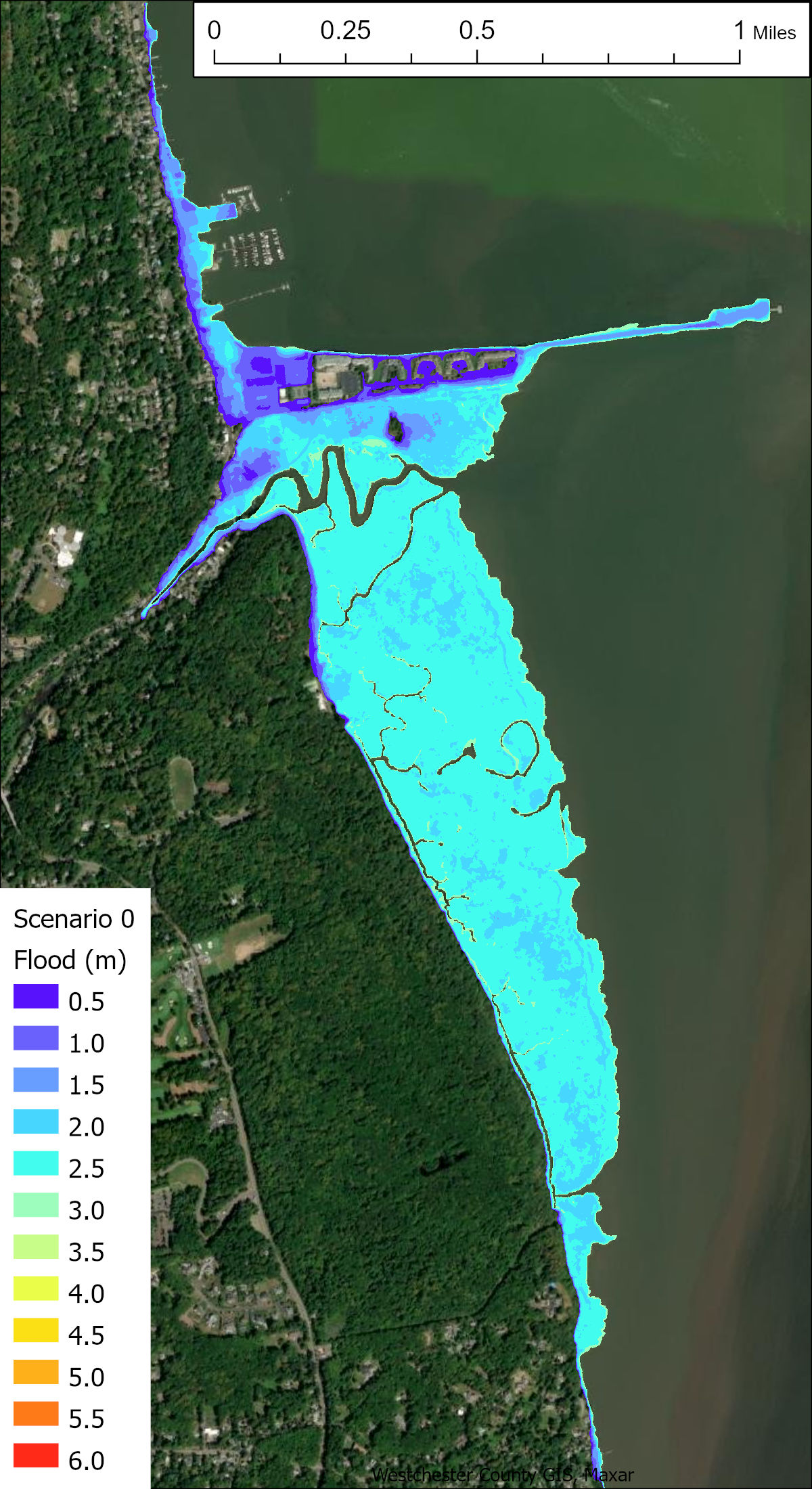


Figure 1. Maximum flood map of Piermont during Sandy with (left) and without (middle) the marsh.

1% annual chance flood map of Piermont for 2020 (right). (ArcGIS basemap credit: Westchester County GIS, Maxar).


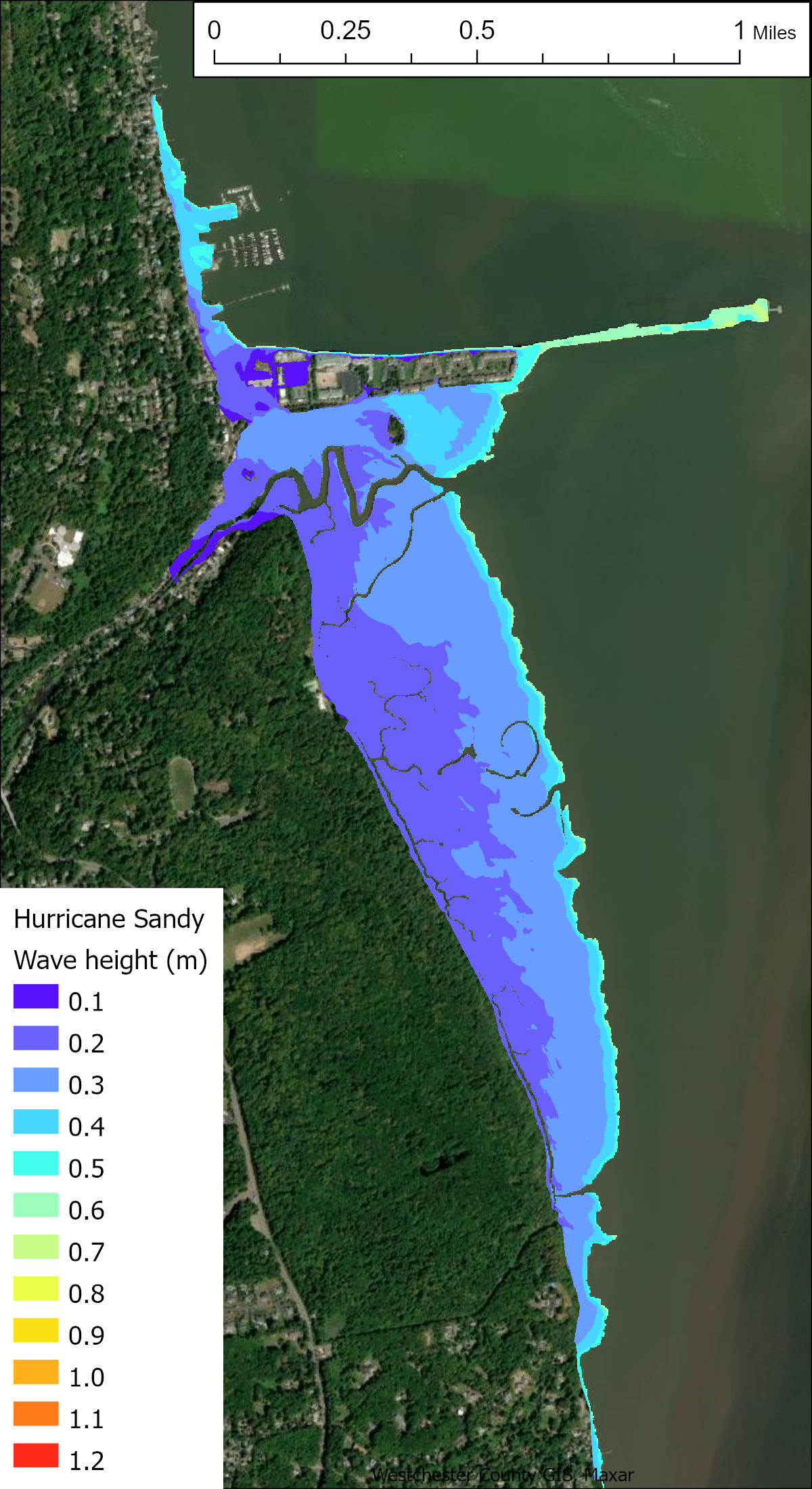

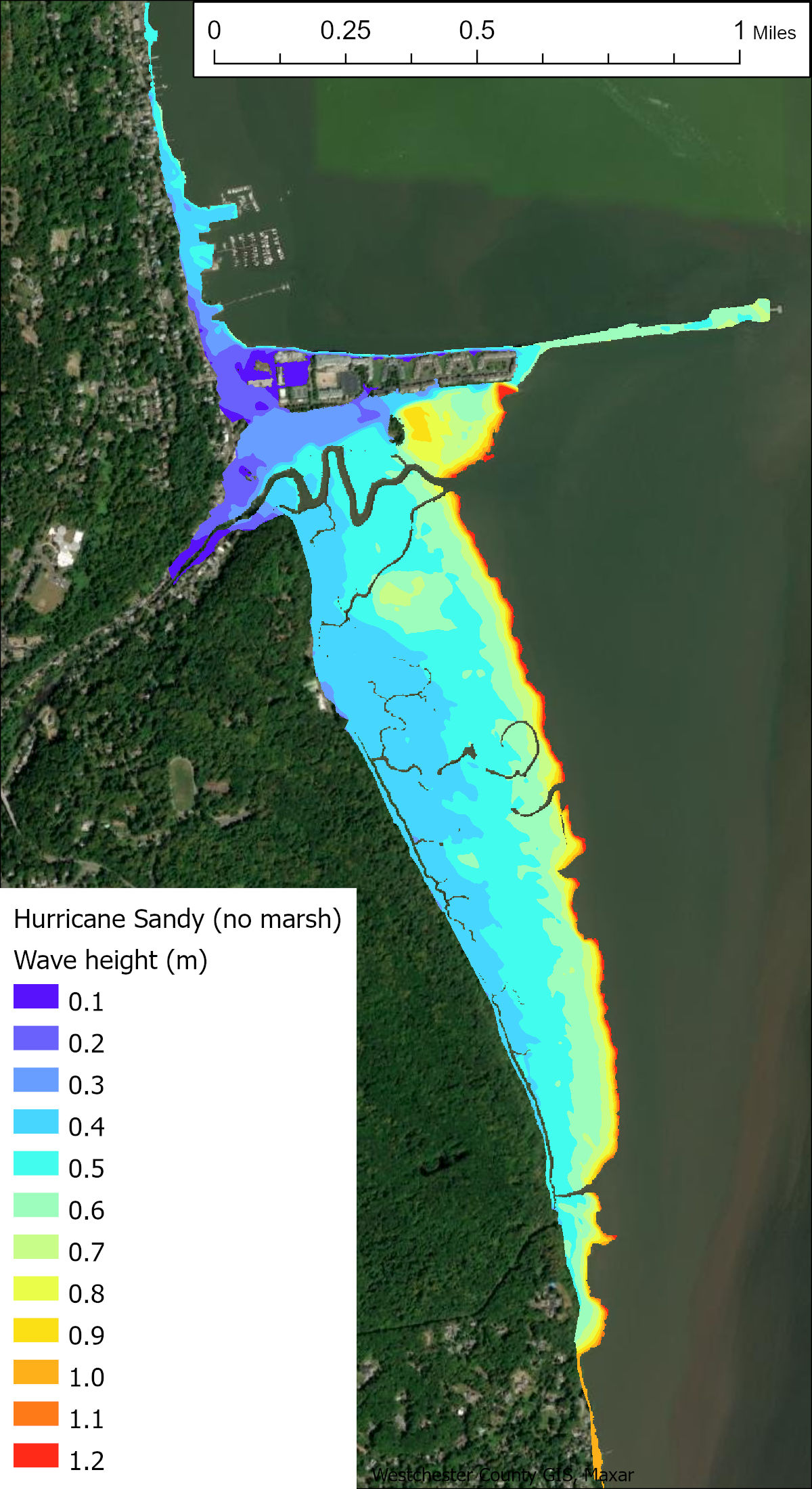

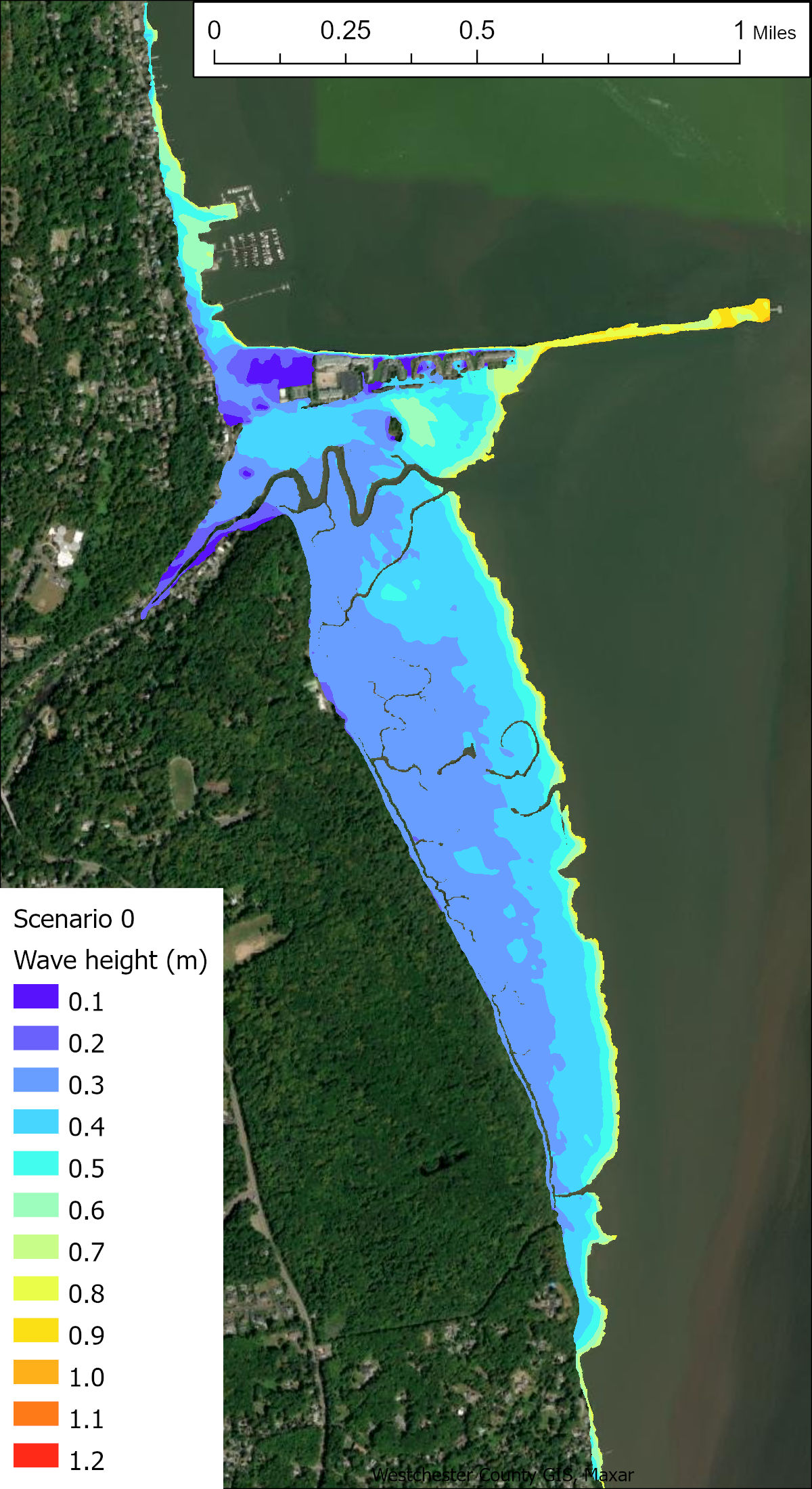


Figure 2. Maximum wave map of Piermont during Sandy with (left) and without (middle) the marsh.

1% annual chance flood map of Piermont for 2020 (right). (ArcGIS basemap credit: Westchester County GIS, Maxar).

Summary of Economic Analysis

Due to lack of consensus on the valuation of coastal wetlands for reducing flood- and wave-induced residential structural loss, we present our economic analysis in terms of several metrics that measure the value of coastal wetlands. As shown in the following Table 2, Relative Avoided Loss (RAL) has been used in several studies, including NAR17^1^, LAT19^2^, SHE21a^6^, and SHE21b^7^. Marginal wetland value, which appears to be comparable to the UMV (Unit Marsh Value) of this study, was used by SC20^3^. However, It should be noted that while NAR17, SHE21a, and SHE21b used a dynamic surge-wave model to simulate the structural losses with and without wetlands, LAT19 and SC20 used linear regression method without dynamic model results for their studies.

| **Scenario** | **Piermont Marsh** | **TSL ($M)** | **RSL (%)** | **TAL ($M)** | **RAL (%)** | **Flooded**  **Marsh (km^2^)** | **UMV ($/m^2^)** |
| --- | --- | --- | --- | --- | --- | --- | --- |
| Sandy | CC: Current Condition | 11.93 | 2.06 | 0.9 | 7.56 | 1.05 | 0.85 |
| Sandy | None | 12.83 | 2.21 |  |  |  |  |
| Black Swan | CC | 18.79 |  |  | 1.46 |  | 0.24 |
| Black Swan | None | 19.06 |  |  |  |  |  |
| 1% Event | CC | 18.82 | 3.24 | 2.2 | 11.34 | 1.05 | 2.09 |
| 1% Event | None | 20.95 | 3.61 |  |  |  |  |
| 1 (2020) | Phase 1 | 18.82 | 3.24 |  |  |  |  |
| 2 (2022) | Phase 2 | 18.82 | 3.24 |  |  |  |  |
| 3 (2025) | Phase 3 | 21.40 | 3.69 |  |  |  |  |
| 4 (2050) | Fully restored | 28.13 | 4.85 | 3.57 |  | 1.05 | 3.4 |
| 5 (2050) | CC | 28.13 | 4.85 | 3.57 | 12.67 |  | 3.4 |
| 5+ (2050) | None | 31.70 | 5.46 |  |  |  |  |
| 6 (2100) | None (Marsh Lost) | 63.34 | 10.92 |  |  | 1.4 |  |
| 6+ (2100) | Marsh growth with high accretion rate | 60.41 | 10.41 | 2.93 | 4.85 | 1.05 | 2.87 |

Table 2. Total Structural Loss (TSL), Relative Structural Loss (RSL=TSL/total property value in 2017), Total Avoided Loss (TAL), Relative Avoided Loss (RAL), and Unit Marsh Value (UML) of the Piermont Marsh for all scenarios. TAL=TSL without marsh – TSL with marsh, RAL=(TSL without marsh/TSL with marsh) – 1, UMV=TAL/total marsh area, total marsh area=345 acres=1.4km^2^, total flooded area changes with the scenarios.

| **Feature** | **Estimate** | **Standard Error** | **t-statistic** | **p-value** |
| --- | --- | --- | --- | --- |
| Intercept | -742,765,850.63 | 194,976,538.52 | -3.81 | 3.22E-04 |
| Flooded Wetland Area | -6.48 | 3.27 | -1.98 | 5.22E-02 |
| Total At-Risk Structural Value | 0.59 | 0.05 | 10.88 | 5.26E-16 |
| Total Wavecrest Volume | 2.67 | 0.53 | 5.01 | 4.80E-06 |

Table3. NJ county scale structural losses regression model estimates.

Structures in Piermont Village Figure 3 below below shows the building footprints in Piermont Village.


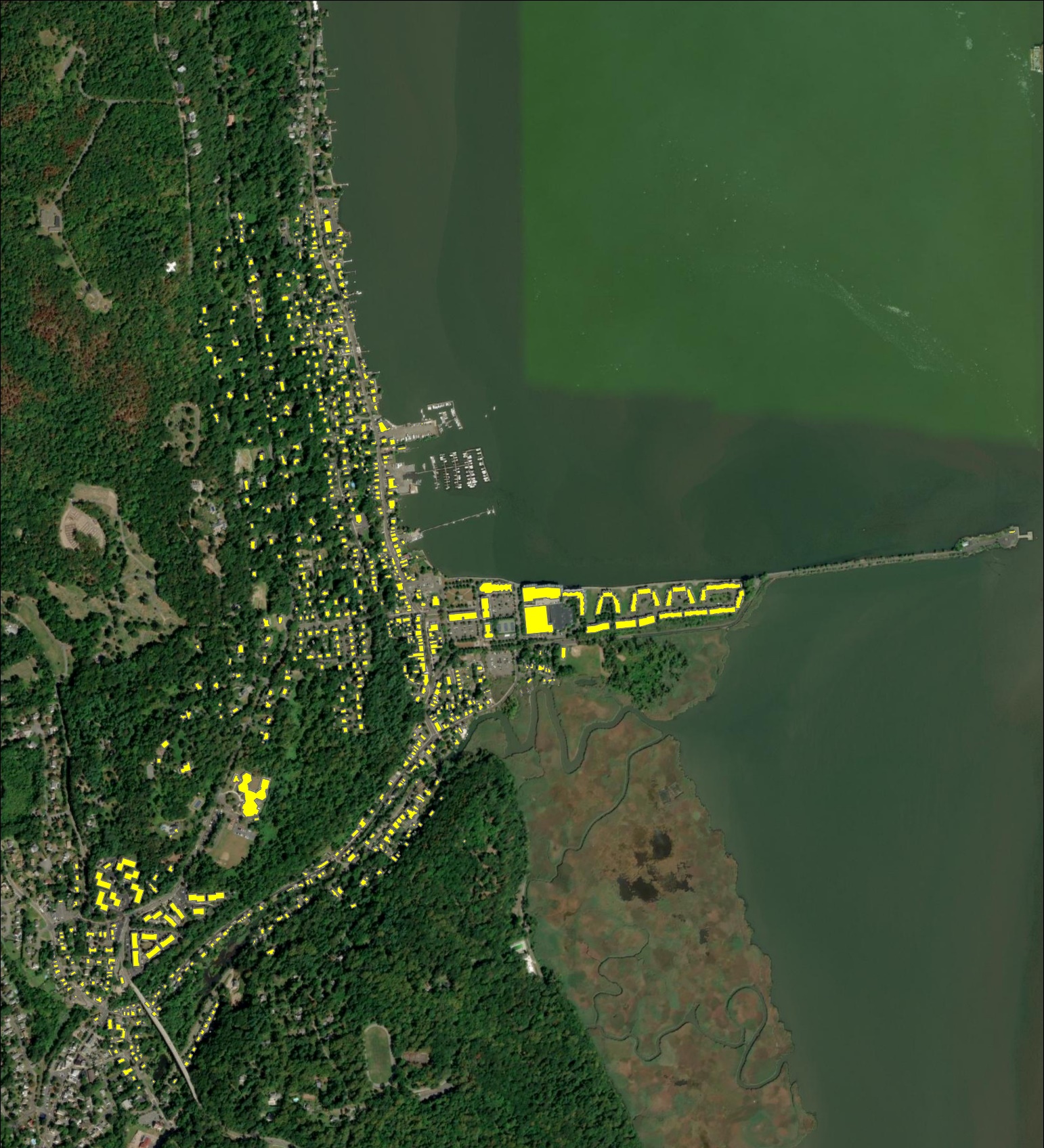


Figure 3. Building footprints (658 total) in Piermont Village (Microsoft Building Footprints, <https://www.microsoft.com/en-us/maps/building-footprints>). (ArcGIS basemap credit: Westchester County GIS, Maxar).
